# Detoxification Mechanism of 8,8-Dimethyl-3-[(*R*-phenyl)amino]-1,4,5(8*H*)-naphthalentrione Derivatives by *Botrytis cinerea*

**DOI:** 10.3390/molecules24030544

**Published:** 2019-02-02

**Authors:** Leonora Mendoza, Marcela Vivanco, Ricardo Melo, Paulo Castro, Ramiro Araya-Maturana, Milena Cotoras

**Affiliations:** 1 Laboratorio de Micología, Facultad de Biología y Química, Universidad de Santiago de Chile, Alameda 3363, Estación Central, Santiago 9160000, Chile; paulo.castro@usach.cl; 2 Núcleo de Química y Bioquímica, Facultad de Estudios Interdisciplinarios, Universidad Mayor, Camino La Pirámide 5750, Huechuraba, Santiago 8580000, Chile; marcela.vivanco@mayor.co (M.V.); ricardo.melo@mayor.cl (R.M.); 3 Instituto de Química de Recursos Naturales and Programa de Investigación Asociativa en Cáncer Gástrico, Universidad de Talca, casilla 747, Talca 3460000, Chile; raraya@utalca.cl

**Keywords:** naphthalentrione derivatives, *Botrytis cinerea*, ABC pump efflux, biotransformation

## Abstract

The effect of 8,8-dimethyl-3-[(*R*-phenyl)amino]-1,4,5(8*H*)-naphthalentrione derivatives (compounds **1**–**13**) on the mycelial growth of *Botrytis cinerea* was evaluated. The fungitoxic effect depended on the substituent and its position in the aromatic ring. Compounds substituted with halogens in meta and/or para positions (compounds **3**, **4**, **5** and **7**), methyl (compounds **8** and **9**), methoxyl (compounds **10** and **11**), or ethoxy-carbonyl groups (compound **12**) presented higher antifungal activity than compound **1**, which had an unsubstituted aromatic ring. In addition, compounds with halogens in the ortho position, such as compounds **2** and **6**, and a substitution with an acetyl group in the para position (compound **13**) were less active. The role of the ABC efflux pump Bctr B-type as a defense mechanism of *B. cinerea* against these naphthalentrione derivatives was analyzed. This pump could be involved in the detoxification of compounds **2**, **6**, and **13**. On the contrary, this mechanism would not participate in the detoxification of compounds **1**, **7**, **9** and **12**. Finally, the biotransformation of compound **7** by *B. cinerea* was studied. A mixture of two biotransformed products was obtained. One of them was compound **7A**, which is reduced at C1 and C4, compared to compound **7**. The other product of biotransformation, 7B, is oxidized at C7.

## 1. Introduction

*Botrytis cinerea* is a common phytopathogenic fungus that causes serious pre- and post-harvest diseases in at least 200 plant species. The broad host range of *B. cinerea* results in great economic losses not only during growth but also during storage and transport [1,2].

This fungus is able to defend itself against toxic compounds through drug efflux transporters [3]. The participation of two groups of protein families has been described: MFS (Major Facilitators Super-Family) and efflux pump ABC transporters (ATP-binding cassette) [4]. ABC efflux pumps are proteins found predominantly in the plasma membrane or in intracellular organelles such as the endoplasmic reticulum, mitochondria and peroxisomes [5]. These pumps can transport against a gradient a wide variety of endogenous toxic agents, such as phytoalexins, antibiotics, and fungicides [3]. In addition, it has been shown that through an ABC efflux pump, *B. cinerea* was able to establish a system of defense against phenazine, since mutants that do not express the ABC efflux pump B are more sensitive to the antibiotic [6]. A similar study was reported in azole-type fungicides where the *bcatrD* gene encoding the ABC efflux pump is involved in the generation of resistance to this fungicide [7].

Additionally, an alternative detoxification mechanism used by *B. cinerea* is the chemical modification or biotransformation of toxic compounds [8]. It has been reported that this fungus can biotransform various families of compounds, such as steroids, flavonoids, monoterpenes, and sesquiterpenes, among others. These modifications are carried out by enzymes, such as hydroxylases, oxygenases, or reductases, producing generally hydroxylations, epoxidations, oxidations, or reductions of the molecules [9,10,11,12,13].

It has been reported that quinone-derivate compounds, such as natural or synthetic naphthoquinones or anthraquinones, exhibit important biological activities, including antibacterial, antifungal, antiparasitic, antiviral, and antitumor activities [14,15,16,17,18,19].

Mendoza et al. described the effect of a set of synthetic structurally related tricyclic hydrocompounds [9,10-dihydroxy-4,4-dimethyl-2,3,5,8-tetrahydroanthracene-1(4*H*)-one and 9,10-dihydroxy-4,4-dimethyl-5,8-dihydroanthracene-1(4*H*)-one derivatives] and tricyclic compounds [4,4-dimethylanthracene-1,9,10(4*H*)-trione derivatives] on the mycelial growth of the fungus *B. cinerea* [20]. In general, the anthra compounds presented higher antifungal activity than the anthrahydro compounds, suggesting that the structure of the anthra compounds is important in the antifungal effect on *B. cinerea* [20].

However, the mechanism used by *B. cinerea* to defend itself from these compounds is still unknown. In the present work, the antifungal activity of 13 8,8-dimethyl-3-[(*R*-phenyl)amino]-1,4,5(8*H*)-naphthalentrione derivatives (compounds **1**–**13**) against *B. cinerea* was evaluated, and the role of the ABC efflux pump B-type as a defense mechanism of *B. cinerea* against antifungal synthetic naphthalentrione derivatives was analyzed. Also, the biotransformation of compound **7** was assessed.

## 2. Results and Discussion

### 2.1. Determination of the Effect of the 8,8-Dimethyl-3-[(R-phenyl)amino]-1,4,5(8H)-naphthalentrione Derivatives on the Mycelial Growth of Botrytis cinerea

**Figure 1:**
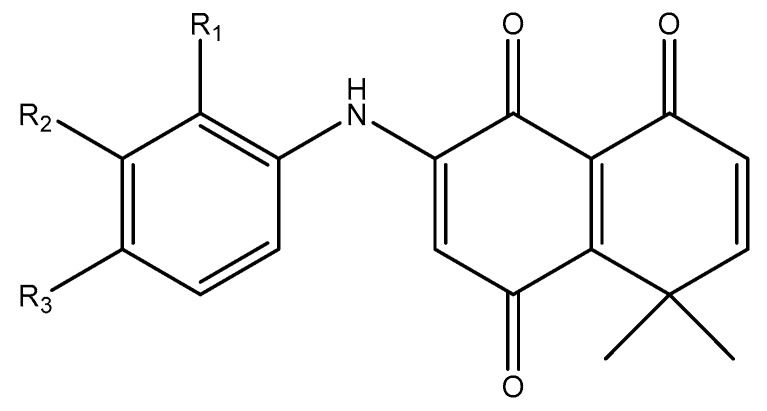
Derivatives of 8,8-dimethyl-3-[(*R*-phenyl)amino]-1,4,5(8*H*)-naphthalentrione used in this study. The different compounds were numbered **1**–**13**.

In this work, the effect of a series of synthetic naphataletriones derived from 8,8-dimethyl-3-[(*R*-phenyl)amino]-1,4,5(8*H*)-naphthalentriones (compounds **1**–**13**) on *B. cinerea* was studied. The basic structural feature of these compounds consists of a naphthalentrione system with an aromatic amine substitution in position 3 (C3). The aromatic ring has several substituents in different positions, as seen in Figure 1.

**Figure 2:**
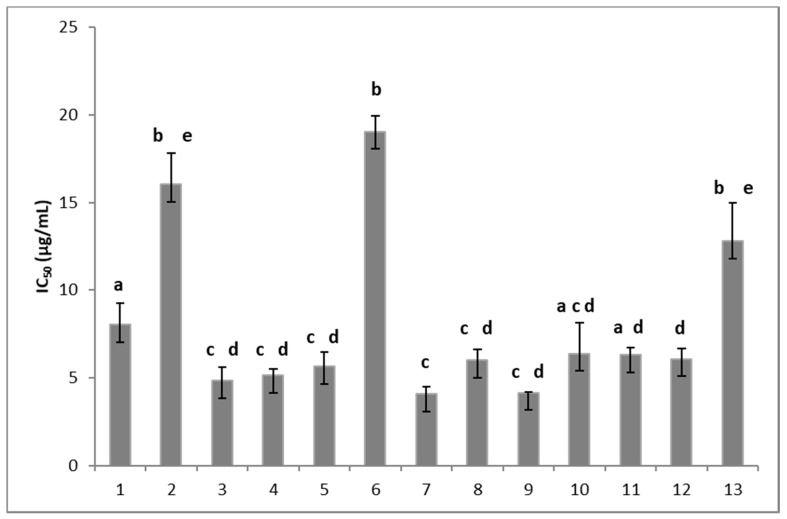
Effect of the 8,8-dimethyl-3-[(*R*-phenyl)amino]-1,4,5(8*H*)-naphthalentrione derivatives on the mycelial growth of *Botrytis cinerea.* The number on the x-axis indicates the tested compound. Each column represents the mean ± standard deviation of three independent experiments. Different letters indicate that the means are significantly different at *p* < 0.05.

The fungitoxicity of these compounds was assessed using the radial growth test on malt-yeast extract agar as described by Mendoza et al. [20]. The effect of 13 synthetic compounds on the mycelial growth of B05.10 isolate from *B. cinerea* in solid media was determined at 72 h of incubation and it was expressed in IC_50_ (μg/mL). Figure 2 shows that all the compounds were fungitoxic, inhibiting the mycelial growth of *B. cinerea*; however, some differences in the fungitoxic effect were observed. Indeed, compounds substituted with halogens in the meta and/or para positions (compounds **3**, **4**, **5**, and **7**), a methyl group (compounds **8** and **9**), methoxyl groups (compounds **10** and **11**), and an ethoxy-carbonyl group (compound **12**) presented the highest antifungal activity, with IC_50_ values of about 5 µg/mL. Compound **1** with an unsubstituted aromatic ring showed an IC_50_ value of 8 μg/mL. It should be noted that compounds that present a substitution with halogens in the ortho position, such as compounds **2** and **6**, and a substitution with an acetyl group in the para position (compound **13**) were less active, with an IC_50_ value higher than 13 μg/mL. Therefore, the fungitoxic effect would depend on the substituent and its position in the aromatic ring.

From these results, it can be concluded that the antifungal effect against *B. cinerea* would be favored when the aromatic ring presents substitutions in the para position, except for the acetyl group substitution. These results are in agreement with previous reports, which showed that chlorophenyl derivates in the para position presented a higher antifungal activity than those substituted in the meta position [21]. On the other hand, the compound 2-methoxy-1,4-naphtoquininone obtained from *Impatiens balsamina* presents antifungal activity against four strains of *Candida albicans* and *Aspergillus niger*. The incorporation of an arylamino, arylthiol group, or halogen atoms in the 1,4-naphthoquinone structures increased the effectiveness of the biological activity [22].

### 2.2. Evaluation of the Effect of the 8,8-dimethyl-3-[(R-phenyl)amino]-1,4,5(8H)-naftalentrione Derivatives on the Germination of B. cinerea

**Figure 3:**
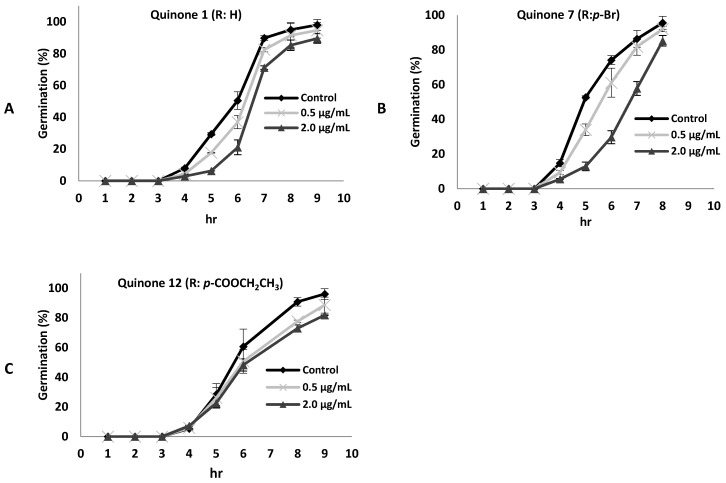
Effect of compounds **1** (**A**), **7** (**B**), or **12** (**C**) at different concentrations on *B. cinerea* germination. Data represents mean ± standard deviation of three independent experiments.

It should be noted that conidial germination is an important early step in the infection process of the fungus. Therefore, the effect of some 8,8-dimethyl-3-[(*R*-phenyl)amino]-1,4,5(8*H*)-naphthalentrione derivatives on conidial germination was evaluated. The compounds tested were **1**, **7**, and **12**, as shown in Figure 3. It was observed that *B. cinerea* germination was slightly delayed in the presence of compound **7** at 2 µg/mL. However, after eight hours of incubation, a percentage of germination similar to that of the control was reached (Figure 3A). On the other hand, compounds **1** or **12** did not have a significant effect on germination (Figure 3B,C). It should be mentioned that these compounds did not provoke morphological changes in the germ tubes (data not shown).

It has been reported that antifungal compounds that inhibit the mitochondrial electron transport chain are more active on conidial germination than on mycelial growth [23]. On the contrary, compound **7** presented a higher fungitoxic effect on mycelial growth than on conidial germination, and this compound did not affect oxygen consumption in the germinating conidia of *B. cinerea* (result not shown).

### 2.3. Study of the Defense Mechanism of B. cinerea against Specific Compounds

It has been described that in *B. cinerea*, ABC efflux transporters are involved in the detoxification of stilbenes, isoflavones, coumarins, and sesquiterpenes as a defense mechanism and they also contribute to fungal resistance to various antibiotics and commercial fungicides [6].

To evaluate if this detoxification mechanism confers protection to *B. cinerea* against the compounds, a mutant strain that does not express the ABC pump B-type expulsion (B05.10ΔBcAtrB) was used. The compounds used in this study were selected according to their effect on *B. cinerea* mycelial growth and were divided into two groups: compounds that showed the highest IC_50_ (compounds **2**, **6**, and **13**) and those that presented the lowest IC_50_ (compounds **1**, **7**, **9**, and **12**). Table 1 shows the effect of these compounds on the mycelial growth of wild-type and mutant strains.

It was found that B-type ABC efflux pumps would be involved in the detoxification from compounds **2**, **6**, and **13**, since the mutant strain presented higher sensitivity to these compounds than the wild-type strain. On the contrary, this mechanism would not participate in the detoxification of compounds **7** and **12** because the effect of these compounds on the mycelial growth of both strains did not show significant differences. Furthermore, the mutant strain exhibited lower sensitivity to compounds **1** and **9** than the wild-type strain suggesting that this B-type ABC efflux pump would not be involved in the detoxification process.

On the other hand, in *B. cinerea,* it was shown that in addition to the detoxification mechanism mediated by transport proteins, biotransformation of the toxic compounds could occur [24]. The efflux pump BcAtrB would participate as the first line of defense, avoiding the accumulation of the antibiotic 2,4-diacetylphloroglucinol in the hyphae. On the other hand, an extracellular laccase would degrade this antibiotic [24].

**Figure 4:**
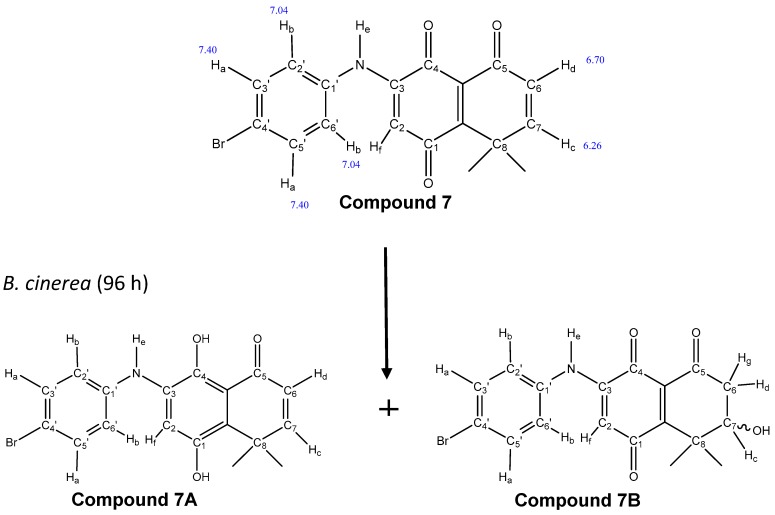
Proposed structures (**7A** and **7B**) of the biotransformed compounds by the fungus *B.cinerea*.

In order to determine if these compounds can be biotransformed by *B. cinerea*, compound **7** was incubated with the wild-type strain for 96 h. The culture medium was extracted with ethyl acetate and analyzed by TLC. In the chromatogram, several products were observed. To characterize the majority product, a new separation by preparative TLC was carried out. The product obtained turned out to be a mixture of two biotransformed products, called **7A** and **7B** (Figure 4). The ^1^H-NMR spectrum signals of the mixture (**7A** and **7B**) and of compound **7** are shown in Table 2.

In order to obtain information on the modification of the structure produced by the fungus in compound **7**, the spectrum of the pure compound was compared with that of the mixture (spectrum no shown).

The presence of signals corresponding to the aromatic ring (at 7.40 ppm) in the structure of compound **7** [14] were also seen in the spectrum of the mixture, suggesting that the carbons of the aromatic ring of compound **7** were not modified by the fungus. Supporting this observation, the signal corresponding to adjacent aromatic protons (7.04 ppm) appeared with the same displacement as that reported by Martinez-Cifuentes et al. [25]. Furthermore, the signals corresponding to the protons on C6 and C7, at 6.7 and 6.26 ppm, respectively, were the same signals present in compound **7** (Figure 4). Therefore, it was related to the absence of modification of the enone fraction of compound **7**. However, downfield in the spectrum, a new signal appeared at 13.5 ppm with the integration for two protons. This signal may correspond to protons of OH groups by reduction of the C1 and C4 carbonyl groups of the quinone system. The 13.5 ppm displacement suggested that the OH on C4 could form intramolecular hydrogen bonds with the carbonyl of C5. Moreover, upfield in the spectrum, there were three important new chemical signals (4.24, 2.34, 2.24 ppm), which suggested that at least one of the compounds in the mixture presents changes in the enone fraction of compound **7**. The emergence of a triplet at 4.24 ppm, corresponding to proton “c” coupling with the C6 protons “d” and “g”, may be attributable to the presence of the OH group bonded on C7. On the other hand, the doublet at 2.24 ppm corresponded to proton “g” coupling with proton “d” and proton “c” (2*J* = 11 Hz and 3*J* = 6 Hz, respectively). Proton “d” was seen as a doublet of doublets at 2.34 ppm, coupling with proton “g” and proton “c” of C-7 (2*J* = 11 Hz and 3*J* = 7 Hz, respectively, data no shown).

The emergence of new important signals seen in the spectrum (Table 2, compound **7B**) suggested that in the mixture there were two compounds, which would correspond to the products of the biotransformation of compound **7** produced by the fungus. One of them corresponded to compound **7A**, which was reduced in C1 and C4, compared to C1 and C4 of compound **7**. The other product of biotransformation, called **7B**, was oxidized in C7 (Figure 4). These results were complemented with a mass spectrometry analysis of the mixture, in which the presence of a molecular ion at *m/z* 374 was consistent with the molecular formula C_18_H_16_BrNO_3_ (compound **7A**). Furthermore, the presence of a second molecular ion found as [M + H]^+^ at *m/z* 390 was consistent with the molecular formula C_18_H_16_BrNO_4_ (compound **7B**).

So far, it was known that *B. cinerea* is able to modify compounds from toxic to products with lower toxicity through enzymes, such as hydroxylases, epoxidases, or reductases [8,26,27,28,29]. Hargreavfs et al. [30] reported that *B. cinerea* was able to reduce the carbonyl group present in the phytoalexin wierona acid after three days of incubation [30]. The hydroxylated compound was less active against *B. cinerea* than the phytoalexin.

On the other hand, it was shown that *B. cinerea* biotransforms the antifungal compound diisophorone into hydroxylated products that are inactive against *B. cinerea* [28]. Additionally, it has been shown that the presence of several hydroxyl groups reduces the antifungal activity against *B. cinerea* of compounds such as flavones [31] or stilbenes [32].

On the basis of the above description, it may be hypothesized that biotransformed products would be less active than compound **7** because they are hydroxylated.

## 3. Materials and Methods

### 3.1. Naphthalentrione Derivatives Used

The 8,8-dimethyl-3-[(*R*-phenyl)amino]-1,4,5(8*H*)-naphthalentrione derivatives used in this study were synthetized as described by Martinez-Cifuentes et al. (2014) [25]. The chemical structures of the compounds (**1**–**13**) are shown in Figure 1.

### 3.2. Characterization of Antifungal Activity

#### 3.2.1. Fungal Isolate and Culture Conditions

In this study, the *B. cinerea* strain B05.10 was used and maintained on malt-yeast extract agar slants (2% (*w*/*v*) malt extract, 0.2% (*w*/*v*) yeast extract, and 1.5% (*w*/*v*) agar) at 4 °C. The fungus was cultivated in the dark on malt-yeast extract agar medium or soft agar medium (2% (*w*/*v*) malt extract, 0.2% (*w*/*v*) yeast extract, and 0.6% (*w*/*v*) agar). Also, the following liquid minimum medium was used: KH_2_PO_4_ (1 g/L), K_2_HPO_4_ (0.5 g/L), MgSO_4_·7H_2_O (0.5 g/L), KCl (0.5 g/L), FeSO_4_·7H_2_O (0.01 g/L), pH 6.5. As a source of nitrogen and carbon, ammonium tartrate (25 mmol/L) and glucose (1% (*w*/*v*) were used. In addition, the mutant strain (B05.10ΔBcAtrB), in which the gene encoding the pump B efflux is disrupted, was also used [6]. This mutant was kindly provided by Dr. Jan van Kan (Wageningen University, Wageningen, the Netherlands).

#### 3.2.2. Evaluation of Conidial Germination and Mycelial Growth of *B. cinerea* in the Presence of Different Compounds

The activity of different naphthalentrione derivatives on the mycelial growth of *B. cinerea* was assessed in vitro as described by Mendoza et al. [33]. All the experiments were carried out at least in triplicate and with adequate controls (solvent and positive controls). The results of the antifungal effect were expressed as IC_50_, determined by the inhibition of radial growth (percent control) at different compound concentrations after 48 h of incubation by the log dose/probit regression line method. Each experiment was performed at least in triplicate. Also, the effect of naphthalentrione derivatives on conidial germination was determined as described by Cotoras et al. [34]. Conidial germination assays were carried out on microscope slides (Merck, Santiago, Chile) coated with soft agar medium (2 mm thickness). Compounds, dissolved in dichloromethane, were added at a final concentration of 40 μg/mL. Dichloromethane was allowed to evaporate prior to inoculation. The slides were inoculated with dry conidia obtained from sporulated mycelia (one-week old), placed in a humid chamber (90% relative humidity), and incubated in the dark at 22 °C for 7 h. Conidial germination was determined directly on the slides at 1 h intervals. The percentage of germination was estimated by counting the number of germinated conidia in five microscope fields, each containing approximately 40 conidia. It was determined that conidia had germinated when the germ tube length was equal or greater than the conidial diameter. Each experiment was performed at least in triplicate.

#### 3.2.3. Effect of Compound **7** on Oxygen Consumption of *B. cinerea* Conidia

Oxygen consumption was determined polarographically at 25 °C with a Hansatech oxygen electrode, using germinating conidia in a total volume of 1 mL, as described by Cotoras et al. [34]. To obtain conidia in suspension, Murashige and Skoog’s basal medium at 4.4 g/L was added to Petri dishes containing the conidia. The conidia were harvested by scraping with a sterile spatula. To eliminate mycelia, the suspension was filtered through glass wool. Conidia concentration was adjusted to 1 × 10^7^ conidia/mL with liquid minimum medium in the presence of 2% (*w*/*v*) glucose. Conidia were incubated for 2 h at 22 °C. A measurement of basal oxygen consumption was made for 2 min in the same liquid minimum medium. After that time, carbonyl cyanide m-chlorophenylhydrazone (CCCP, 0.05 mM), KCN (10 mM), or compound **7** at final concentration of 2, 4, and 8 ppm were added. Oxygen consumption was determined for eight more minutes. As a control, KCN and CCCP were used as inhibitor or uncoupler of the respiratory chain, respectively.

### 3.3. Analysis of the Defense Mechanism of Botrytis cinerea against the Specific Naphthalentrione Derivatives

#### 3.3.1. Role of the ABC Efflux Pumps in the Defense of *B. cinerea* in the Presence of Compounds **1**, **2**, **6**, **7**, **9**, **12**, and **13**

In this study, the B05.10 strain, (wild-type) and a mutant strain (B05.10ΔBcAtrB) were used. The mutant strain is not able to express the efflux pump ATP-binding cassette (ABC) type-B [6]. The effect of the compounds on the mycelial growth of the mutant strain was determined as previously but in the presence of 70 mg/L of hygromycin B. All assays were performed in triplicate, and IC_50_ was expressed in µg/mL.

#### 3.3.2. Biotransformation Assay

Erlenmeyer flasks containing 10 mL of yeast-malt medium and 0.1% (*v*/*v*) Tween-20 were inoculated with a conidial suspension of isolate B05.10 (1 × 10^6^ conidia/mL). The cultures were incubated in a rotary shaker at 180 rpm and 22 °C for 72 h. After this time, the culture medium was removed. The mycelium was transferred to a 50 mL Erlenmeyer flask containing 10 mL of liquid minimal medium supplemented with 1% (*w*/*v*) glucose and 0.1% (*v*/*v*) Tween-20 in the presence or absence of compound **7** at 25 µg/mL and incubated at 22 °C for 62 and 96 h at 180 rpm.

The following controls were used: cultures containing the fungus in the absence of the compound, cultures containing the compound in the absence of fungi, and cultures containing only culture media in the absence of both fungus and compound.

After the incubation time, the mycelium was separated from the culture medium by vacuum filtration and washed with 10 mL of ethyl acetate. The filtrate culture was acidified to pH 3 and extracted with ethyl acetate. The aqueous phase was discarded, and anhydrous sodium sulfate as a drying agent was added to the organic phase for 4 or 12 h. Then, the solvent was evaporated in a rotary evaporator.

#### 3.3.3. Purification and Characterization of Biotransformation Products

In total, 40 mg of extract containing the biotransformation products obtained from the culture media was purified on semi-preparative thin-layer chromatography (TLC) plates pre-coated with silica gel 60 F-254 (0.5-mm-thick plates; Merck, Santiago, Chile), using hexane/ethyl acetate 3:2 as the elution system. The identification of biotransformation products was made by nuclear magnetic resonance (^1^H-NMR) on a BRUKER Avance 400 spectrometer (1 H, 400.13 MHz, Bruker, Billerica, MA, USA). Chemical shifts (in ppm) for ^1^H were reported relative to Me_4_Si (TMS). Mass spectra of the biotransformed compounds were acquired using an electrospray mass spectrometer ion-trap Esquire model 4000 ESI-IT (Bruker, Billerica, MA, USA). Control of the spectrometer was performed using the programs quire Control 5.2. A stock solution was prepared adding 200 µL of acetonitrile to the sample. From this solution, a working solution was prepared consisting of 50 μL of stock solution, 100 mL of acetonitrile, and 50 L of water; formic acid was added to a final concentration of approximately 0.3% *v*/*v*. Later, ca. 50 mL of working solution was injected using a syringe pump at a flow rate of 2.5 μL/min. The ionization process was performed by electrospray 4000V-aided nebulizer nitrogen gas at a temperature of 300 °C, a pressure of 10 psi, and a flow rate of 5 L/min (Bruker, Billerica, MA, USA). Spectra acquisition (Bruker, Billerica, MA, USA) was performed in positive and negative polarity.

### 3.4. Experimental Design and Statistical Analyses

The antifungal activities of the different compounds against *B. cinerea* were analyzed with a one-way analysis of variance (Prism 5.01, GraphPad Software, San Diego, CA, USA). The means were separated with the least significant difference test (*p* < 0.05).

## Figures and Tables

**Table:** 

COMPOUND NUMBER	R1	R2	R3
**1**	H	H	H
**2**	Cl	H	H
**3**	H	Cl	H
**4**	H	H	Cl
**5**	H	Cl	Cl
**6**	Br	H	H
**7**	H	H	Br
**8**	CH_3_	H	H
**9**	H	H	CH_3_
**10**	H	H	OCH_3_
**11**	H	OCH_3_	OCH_3_
**12**	H	H	COOCH_2_CH_3_
**13**	H	H	COCH_3_

**Table 1:** Effect of compounds **1**, **2**, **6**, **7**, **9**, **12**, and **13** on the mycelial growth of B05.10 and B05.10ΔBcatrB strains.

Compound	IC_50_ (µg/mL)	
	B05.10	B05.10ΔBcAtrB
**1**	8.0 ± 1.2 *	16.3 ± 2.2 *
**2**	16.0 ± 1.8 *	9.4 ± 0.3 *
**6**	19.0 ± 0.9 *	9.8 ± 2.6 *
**7**	4.1 ± 0.4	5.0 ± 0.5
**9**	4.2 ± 0.1	5.6 ± 0.7
**12**	6.1 ± 0.6	5.4 ± 1.5
**13**	12.8 ± 2.2 *	5.8 ± 1.4 *

* IC_50_ value in the wild type and mutant are significantly different (*p* < 0.05).

**Table 2:** ^1^H-NMR data of compound **7** and its biotransformation products (**7A** and **7B**).

Compounds		
7	7A	7B
δ (ppm)	δ (ppm)	δ (ppm)
7.40 d (Ha)	7.40 (Ha)	7.40 (Ha)
7.04 d (Hb)	7.04 (Hb)	7.04 (Hb)
6.7 d (Hd)	6.7 (Hd)	4.24 t (Hc)
6.26 d (Hc)	6.26 (Hc)	2.34 d (Hd)
		2.24 d (Hg)

## References

[1] Dean R., Van Kan J., Pretorius Z., Hammond-Kosack K.E., Di Pietro A., Spanu P., Rudd J., Dickman M., Kahmann R., Ellis J. (2012). The Top 10 fungal pathogens in molecular plant pathology. Mol. Plant Pathol..

[2] Fillinger S., Elad Y. (2016). Botrytis–the Fungus, the Pathogen and Its Management in Agricultural Systems.

[3] De Waard M.A. (1997). Significance of ABC Transporters in fungicide sensitivity and resistance. Pestic. Sci..

[4] Coleman J.J., Mylonakis E. (2009). Efflux in Fungi: La Piece de Resistance. PLoS Pathog..

[5] Stefanato F.L., Abou-Mansour E., Buchala A., Kretschmer M., Mosbach A., Hahn M., Bochet G., Metraux J.P., Schoonbeek H.J. (2009). The ABC transporter *BcatrB* from *Botrytis cinerea* exports camalexin and is a virulence factor on *Arabidopsis thaliana*. Plant J..

[6] Schoonbeek H., Del Sorbo G., De Waard M.A. (2001). The ABC Transporter *BcatrB* affects the sensitivity of *Botrytis cinerea* to the phytoalexin resveratrol and the fungicide Fenpiclonil. Mol. Plant Microbe In..

[7] Hayashi K., Schoonbeek H.J., Sugiura H., Maarten A. (2001). Multidrug resistance in *Botrytis cinerea* as sociated with decreased accumulation of the azole fungicide Oxpoconazole and increased transcription of the ABC transporter gene *BcatrD*. Pestic. Biochem. Phys..

[8] Kretschmer M., Leroch M., Mosbach A., Walker A.-S., Fillinger S., Mernke D., Schoonbeek H.-J., Pradier J.-M., Leroux P., De Waard M.A. (2009). Fungicide-driven evolution and molecular basis of multidrug resistance in field populations of the grey mould fungus *Botrytis cinerea*. PLoS Pathog..

[9] Kovačec E., Regvar M., Van Elteren J.T., Arčon I., Papp T., Makovec D., Vogel-Mikuš K. (2017). Biotransformation of copper oxide nanoparticles by the pathogenic fungus *Botrytis cinerea*. Chemosphere.

[10] Mendoza L., Sepúlveda C., Melo R., Cotoras M. (2015). Characterization of the antifungal activity against *Botrytis cinerea* of sclareol and 13-epi-sclareol, two labdane-type diterpenoid. J. Chil. Chem. Soc..

[11] Ascari J., Diamantino M., Takahashi J., Durán-Patrón R., Hernández-Galán R., Macías-Sánchez A., Collado I. (2011). Biotransformation of bioactive isocaryolanes by *Botrytis cinerea*. J. Natl. Prod..

[12] Aleu J., Collado I. (2001). Biotransformations by *Botrytis* species. J. Mol. Catal. B-Enzym..

[13] Farooq A., Tahara S. (2000). Biotransformation of testosterone and pregnenolone catalyzed by the Fungus *Botrytis cinerea*. J. Nat. Prod..

[14] Fouillaud M., Venkatachalam M., Girard-Valenciennes E., Caro Y., Dufossé L. (2016). Anthracompounds and derivatives from marine-derived fungi: Structural diversity and selected biological activities. Mar. Drugs.

[15] Rahmoun N.M., Boucherit-Otmani Z., Boucherit K., Benabdallah M., Villemin D., Choukchou-Braham N. (2012). Antibacterial and antifungal activity of lawsone and novel naphthocompound derivatives. Med. Maladies Infect..

[16] Tandon V.K., Chhor R.B., Singh R.V., Rai S., Yadav D.B. (2004). Design, synthesis and evaluation of novel 1,4-naphthocompound derivatives as antifungal and anticancer agents. Bioorg. Med. Chem. Lett..

[17] Sasaki K., Hidetomo A., Yoshizaki F. (2002). In vitro antifungal activity of naphthocompound derivatives. Biol. Pharm. Bul..

[18] Kapadia G.J., Azuine M.A., Balasubramanian V., Sridhar R. (2001). Aminonaphthocompounds A novel class of compounds with potent antimalarial activity against *Plasmodium falciparum*. Pharmacol. Res..

[19] Gafne S., Wolfender J.L., Nianga M., Stoeckli-Evans H., Hostettman K. (1996). Antifungal and antibacterial naphthoquinones from Newbouldia laevis roots. Phytochemistry.

[20] Mendoza L., Araya R., Cardona W., Delgado T., Garcia C., Lagos C., Cotoras M. (2005). In vitro sensitivity of *Botrytis cinerea* to anthracompound and anthrahydrocompound derivatives. J. Agr. Food Chem..

[21] Pezet R., Pont V., Daniel M., Purkayastha R.P. (1995). Mode of toxic action of *Vitaceae* stilbenes on fungal cells. Handbook of Phytoalexin Metabolism and Action.

[22] Ryu C., Shim J.Y., Chae M., Choi I.H., Han J.Y., Jung O., Lee J., Jeong S. (2005). Synthesis and antifungal activity of 2/3-arylthio- and 2,3-bis(arylthio)-5-hydroxy-/5-methoxy-1,4-naphthoquinones. Eur. J. Med. Chem..

[23] Slawecki R.A., Ryan E.P., Young D.H. (2002). Novel fungitoxicity assays for inhibition of germination-associated adhesion of *Botrytis cinerea* and *Puccinia recondita* spores. Appl. Environ. Microb..

[24] Schouten A., Maksimova O., Cuesta-Arenas Y., van den Berg G., Raaijmakers J.M. (2008). Involvement of the ABC transporter BcAtrB and the laccase BcLCC2 in defense of *Botrytis cinerea* against the broad-spectrum antibiotic 2,4-diacetylphloroglucinol. Environ. Microbiol..

[25] Martınez-Cifuentes M., Clavijo-Allancan G., Di Vaggio-Conejeros C., Weiss-Lopez B., Araya-Maturana R. (2014). On-Water Reactivity and Regioselectivity of Compounds in C-N Coupling with Amines: Experimental and Theoretical Study. Aust. J. Chem..

[26] Pinedo-Rivilla C., Bustillo A.J., Hernández-Galán R., Aleu J., Collado I. (2011). Asymmetric preparation of antifungal 1-(4′-chlorophenyl)-1-cyclopropyl methanol and 1-(4′-chlorophenyl)-2-phenylethanol. Study of the detoxification mechanism by *Botrytis cinerea*. J. Mol. Catal. B-Enzym..

[27] Farooq A.B., Choudhary M.I., Atta-ur-Rahmana, Tahara S., Baser K.H., Demirci F. (2002). Detoxification of Terpinolene by plant pathogenic fungus *Botrytis cinerea*. Zeitschrift für Naturforsch C.

[28] Daoubi M., Deligeorgopoulou A., Macias J.A., Hernandez H., Hitchcock P.B., Hanson J.R., Collado I.G. (2005). Antifungal activity and biotransformation of diisophorone by *Botrytis cinerea*. J. Agr. Food Chem..

[29] Aleu J., Hanson J.R., Hernández R., Collado I.G. (1999). Biotransformation of the fungistatic sesquiterpenoid patchoulol by *Botrytis cinerea*. J. Nat. Prod..

[30] Hargreavfs J.A., Mansfield J.W., Coxon D.T., Prig K. (1976). Wyerone epoxide as a phytoalexin in *Vicia faba* and its metabolism by *Botrytis cinerea* and *B. fabae* in vitro. Phytochemistry.

[31] Cotoras M., García C., Lagos C., Folch C., Mendoza L. (2001). Antifungal activity on *Botrytis cinerea* of flavonoids and diterpenoids isolated from the surface of *Pseudognaphalium* spp. Bol. Soc. Chil. Quím..

[32] Caruso F., Mendoza L., Castro P., Cotoras M., Aguirre M., Matsuhiro B., Isaacs M., Rossi M., Viglianti A., Antonioletti R. (2011). Antifungal Activity of Resveratrol against *Botrytis cinerea* Is Improved Using 2-Furyl Derivatives. PLoS ONE.

[33] Mendoza L., Cotoras M., Vivanco M., Matsuhiro B., Torres S., Aguirre M. (2013). Evaluation of antifungal properties against the phytopathogenic fungus *Botrytis cinerea* of anthocyanin rich-extracts obtained from grape pomaces. J. Chil Chem Soc..

[34] Cotoras M., Mendoza L., Muñoz A., Yáñez K., Castro P., Aguirre M. (2011). Fungitoxicity against *Botrytis cinerea* of a Flavonoid Isolated from *Pseudognaphalium robustum*. Molecules.

